# 4525 Estimates of Dose Response using the Dixon Up-and-Down Method and BOIN study designs

**DOI:** 10.1017/cts.2020.177

**Published:** 2020-07-29

**Authors:** Charles Gene Minard

**Affiliations:** 1 Baylor College of Medicine

## Abstract

OBJECTIVES/GOALS: The Dixon up-and-down method (U/D), original developed for testing explosives, is especially common in anesthesia research studies. The objective of this research is to compare the performance of the U/D method for obtaining and analyzing sensitivity data with that of the Bayesian Optimal Interval (BOIN) method. METHODS/STUDY POPULATION: A simulation study will compare the performance of the U/D method with the BOIN design. The two study designs offer alternative decision-making algorithms with respect to the dose at which the next experimental unit is treated. These alternative decisions may impact the precision of point estimates of the mean and standard deviation of the effective dose to elicit a response. Transition probability matrices are developed, and maximum likelihood estimates of the unknown parameters assessed for accuracy. For simulation, the effective dose is assumed to be randomly distributed with a known mean and standard deviation. Fixed dose levels are defined, and decisions for what level the next experimental unit should be treated at are defined by the Dixon up-and-down method and the BOIN design. For the U/D method, the stimulus is increased by one level in the absence of a response or decreased if a response occurs from an initial stimulus. A target toxicity probability of 0.50 is used to define the dose escalation or de-escalation rules for the application of the BOIN design. RESULTS/ANTICIPATED RESULTS: A feature of both methods is that the consecutive observations are concentrated about the mean value of the effective dose. However, the BOIN design tends to be more concentrated between these two dose levels. In the presence of severe adverse events, the BOIN design can choose to eliminate doses that are too toxic whereas the U/D design cannot eliminate any dose levels. Transition probability matrices are defined and parameters for the distribution of the effective dose are estimated using maximum likelihood estimation. Mean squared errors for the estimated mean and standard deviations compare the two study designs. DISCUSSION/SIGNIFICANCE OF IMPACT: The BOIN design offers an alternative method for decision-making compared with the U/D method. The BOIN design tends to concentrate dose levels about the mean more than the U/D. This may provide better estimates of the mean and standard deviation of the effective dose for eliciting a response in some circumstances.

